# Breast metastasis from lung cancer: a report of two cases and literature review

**DOI:** 10.7497/j.issn.2095-3941.2014.03.007

**Published:** 2014-09

**Authors:** Li Wang, Shu-Ling Wang, Hong-Hong Shen, Feng-Ting Niu, Yun Niu

**Affiliations:** Department of Breast Cancer Pathology and Research Laboratory; Key Laboratory of Breast Cancer Prevention and Therapy, Tianjin Medical University, Ministry of Education; Key Laboratory of Cancer Prevention and Therapy, Tianjin; Tianjin Medical University Cancer Institute and Hospital, National Clinical Research Center of Cancer, Tianjin 30060, China

**Keywords:** Lung cancer, breast metastasis, immunohistochemistry, thyroid transcription factor-1 (TTF1)

## Abstract

Breast metastasis from extra-mammary malignancy is rare. An incidence of 0.4% to 1.3% has been reported in literature. The primary malignancies that most commonly metastasize to the breast are leukemia, lymphoma, and malignant melanoma. In this report, two cases of pulmonary metastasis to the breast were presented. A 40-year-old female manifested a right breast mass of 2-month duration. After physical examination was performed, a poorly defined mass was noted in the upper outer quadrant of the right breast. Another 49-year-old female manifested right breast mass of 5-day duration. A poorly defined mass was noted in the lower inner quadrant of the right breast. Mammography results also revealed breast cancer. The patients underwent local excision. After histological and immunohistochemical analyses were conducted, a primary lung carcinoma that metastasized to the breast was diagnosed. An accurate differentiation of metastasis to the breast from primary breast cancer is very important because the treatment and prognosis of the two differ significantly.

## Introduction

Primary breast cancer is one of the most common malignancies in adult females, in which metastases to the breast represent approximately 0.4% to 1.3% of malignant tumors in the breast in a clinical series^1^^-^^4^. Despite its rarity, metastatic breast disease is a significant diagnostic clinical problem because its treatment largely differs from that of primary breast cancer. In 1907, Sitzentfrey^5^ firstly published a case of ovarian carcinoma that metastasized to the breast. Thus far, various malignancies, including hematological malignancies, malignant melanoma, lung tumors, renal cell carcinoma, ovarian tumors, thyroid carcinomas, and small bowel carcinoids, have commonly metastasized to the breast^2^^,^^4^^-^^8^. The lung is one of the most common cancer sites in terms of incidence and mortality; however, cases of pulmonary carcinomas metastasizing to the breast have also been published (**Table 1**)^9^^-^^24^.

**Table 1:** Breast metastasis from lung cancer: review of the literature (2003-2013)

References	Year	No. of patients	Age (years)	Gender	Primary tumor type
Bartella *et al*.^9^	2003	1	65	Female	Small cell lung cancer
Masmoudi *et al*.^10^	2003	1	54	Female	Non-small cell lung cancer
Ramar *et al*.^11^	2003	1	56	Male	Non-small cell lung cancer
Yeh *et al*.^12^	2004	1			Lung adenocarcinoma
Shukla *et al*.^13^	2005	2	40	Female	Small cell lung cancer
			42	Female	Small cell lung cancer
Gómez-Caro *et al*.^14^	2006	1	65	Male	Non-small cell lung cancer
Lee *et al*.^15^	2007	5	49	Female	Small cell lung cancer
			49	Female	Small cell lung cancer
			83	Female	Squamous carcinoma
			64	Female	Adenocarcinoma
			58	Male	Large cell carcinoma
Noguera *et al*.^8^	2007	2	51	Female	Oat-cell carcinoma of lung
			41	Female	Anaplastic carcinoma of lung
Williams *et al*.^6^	2007	41			–
Fulciniti *et al*.^16^	2008	1	59	Female	Lung adenocarcinoma
Hsu *et al*.^17^	2008	1	48	Female	Squamous cell lung carcinoma
Wood *et al*.^18^	2008	8			–
Babu *et al*.^19^	2009	3	51	Female	
			69	Female	Small cell lung cancer
			82	Female	Large cell neuroendocrine carcinoma of lung
Lee *et al*.^20^	2010	5			–
Maounis *et al*.^21^	2010	1	73	Female	Pulmonary adenocarcinoma with micropapillary component
Yoon *et al*.^22^	2010	1	42	Female	Non-small cell lung carcinoma
Ko *et al*.^23^	2012	1	47	Female	Lung cancer with micropapillary component
Crona *et al*.^24^	2013	8	44	Female	Lung neuroendocrine tumor
			60	Female	Lung neuroendocrine tumor
			44	Female	Lung neuroendocrine tumor
			28	Female	Lung neuroendocrine tumor
			42	Female	Lung neuroendocrine tumor
			62	Female	Lung neuroendocrine tumor
			45	Female	Lung neuroendocrine tumor
			72	Female	Lung neuroendocrine tumor

Some cases presented breast metastasis from lung tumors. However, no detailed histological classification has been provided. In this report, two patients with breast metastases from pulmonary adenocarcinoma were presented. To our knowledge, this article is the first to report lung cancer cells as single lesions infiltrating into bilateral breast parenchymal tissues. We also performed a differential diagnosis by using a panel of immunohistochemical markers. Written informed consent was obtained from each patient for publication of this case report and accompanying images.

## Case one

A 40-year-old non-smoking nurse was presented to the Tianjin Medical University Cancer Institute and Hospital with a right breast mass of 2-month duration. Physical examination results revealed a mass in the upper outer field of her right breast. The mass was hard and fixed. The skin of her breast was normal. Corresponding superficial lymph nodes were not enlarged. Mammography results revealed right breast cancer. Calcification was not observed. Enhanced chest computed tomography (CT) showed a cavitary mass in the posterior basal segment of the right lower lobe, metastatic nodules in bilateral lungs, and metastatic lymph nodes in the mediastinum and hilum of the right lung; a nodule in the upper outer quadrant of the left breast near the anterior axilla and multiple liver masses were also observed. Magnetic resonance imaging results indicated a nodule in the upper outer quadrant of the left breast near the anterior axilla, suggesting malignancy. Bilateral local excision was performed to remove the rapidly growing breast lesion. Our patient received chemotherapy of paclitaxel and cisplatin and lived for 7 months.

**Figure 1:**
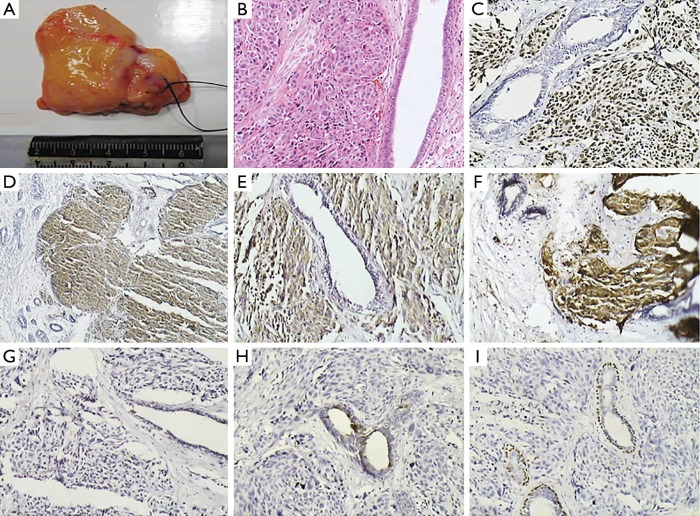
(A) Specimen of the left breast biopsy. Tumor edge is ill-defined and lacks sharp circumscription. Tumor is hard upon palpation. Cut surface is gray white and gray red (0.7 cm × 0.6 cm × 0.5 cm). (B) Spindle cell carcinoma (H&E staining ×100). (C) TTF-1 positive (IHC staining ×100). (D) NSE positive (IHC staining ×100). (E) Syn positive (IHC staining ×100). (F) CgA positive (IHC staining ×100). (G) GCDFP-15 negative (IHC staining ×100). (H) MG negative (IHC staining ×100). (I) p63 negative (IHC staining ×100).

**Figure 2:**
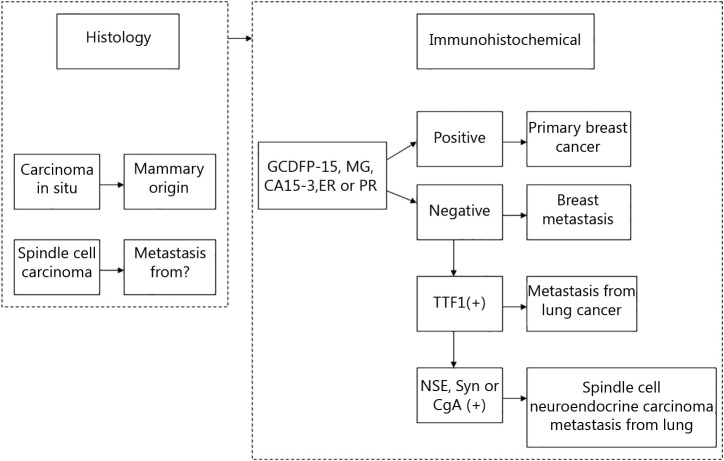
Differential diagnosis of case 1.

Hematoxylin and eosin (H&E)-stained paraffin sections of the breast specimen revealed spindle cell carcinoma. These poorly differentiated tumors were composed of large crowded clusters of cells with moderate to abundant cytoplasm; these tumors also displayed an infiltrative growth pattern. Furthermore, these tumors consisted of densely and solid nests of spindle cells separated by delicate fibrovascular stroma. In some tumors, nuclear streaming occurred. Our differential diagnosis included primary breast carcinoma and metastatic carcinoma from the lungs. The tumor cells demonstrated immunoreactivity for CK7, CK8, thyroid transcription factor-1 (TTF1), neuron-specific enolase (NSE), Syn, CgA, and SOX-2. The neoplastic cells lacked expressions of smooth muscle actin (SMA), p63, calponin, p40, gross cystic disease fluid protein-15 (GCDFP-15), mammaglobin (MG), CA15-3, estrogen receptor (ER), progesterone receptor (PR), c-erb-2, CK 5/6 and CD56. The expressions of Ki67 and p53 showed strong nuclear staining in 50% and 85% of the tumor cells, respectively. No evidence of in situ carcinoma was observed (**Figure 1**). Spindle cell neuroendocrine carcinoma metastasized from the lungs was diagnosed based on histological and immunohistochemical staining patterns (**Figure 2**); this result is consistent with contralateral breast tumor diagnosis.

**Figure 3:**
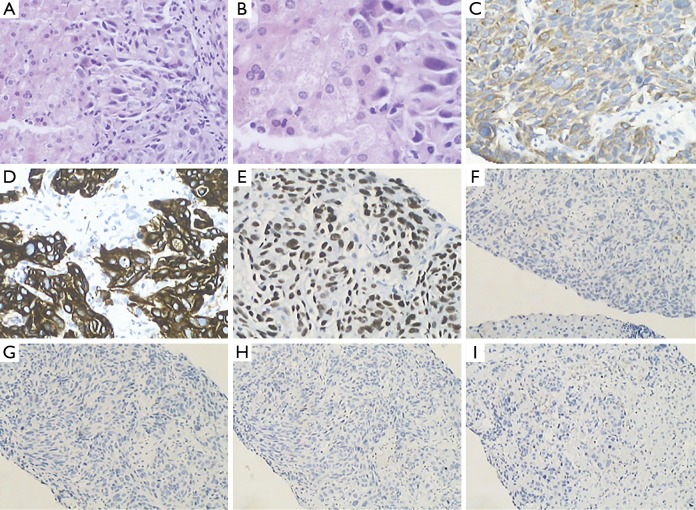
(A) Liver biopsy, carcinoma (H&E staining ×200); (B) Carcinoma. (H&E staining ×400); (C) CK7 positive (IHC staining ×200); (D) CK19 positive (IHC staining ×200); (E) TTF1 positive (IHC staining ×200); (F) GCDFP-15 negative (IHC staining ×100); (G) ER negative (IHC staining ×100); (H) PR negative (IHC staining ×100); (I) CK20 negative (IHC staining ×100).

CT also showed multiple liver masses. Thus, we performed hepatic core needle biopsy. H&E-stained paraffin sections of the hepatic core needle biopsy specimen revealed carcinoma. The tumor cells demonstrated immunoreactivity for CK7, CK19 and TTF1. The neoplastic cells lacked expression of GCDFP-15, ER, PR, c-erb-2, CK20 and CD56 (**Figure 3**). Lung carcinoma metastasis was diagnosed based on histological and immunohistochemical staining patterns.

## Case two

A 49-year-old non-smoking housewife was presented to the Tianjin Medical University Cancer Institute and Hospital with a right breast mass of 5-day duration. Physical examination results revealed a mass in the lower inner field of her right breast. The mass was hard and fixed. The skin of her breast was normal. Corresponding superficial lymph nodes were not enlarged. Enhanced chest CT showed a right breast mass, a nodule in the right upper lung, and metastatic lymph nodes in the mediastinum and right collarbone. Right local excision was performed to remove the rapidly growing breast lesion. The patient initiatively abandoned chemotherapy and lived for 8 months.

**Figure 4:**
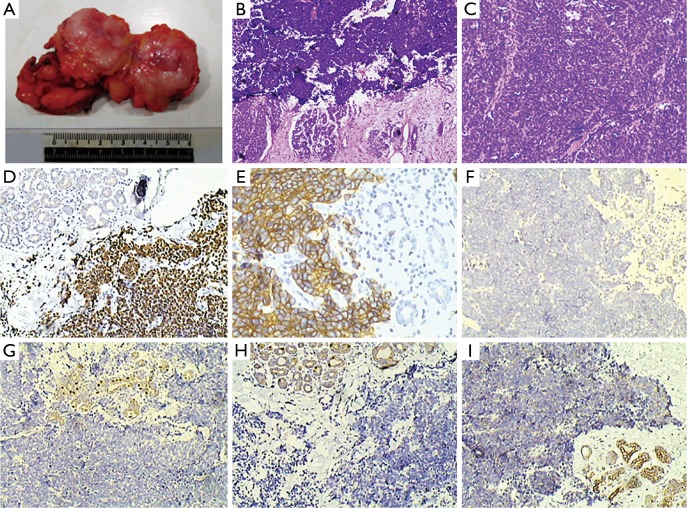
(A) Specimen of the right breast biopsy. Tumor edge is ill-defined and lacks sharp circumscription. Tumor is hard upon palpation. Cut surface is gray white (3.2 cm × 3.0 cm × 2.7 cm). (B) Small cell carcinoma (H&E staining ×40). (C) Small cell carcinoma (H&E staining ×100). (D) TTF-1 positive (IHC staining ×100). (E) CD56 positive (IHC staining ×200). (F) CA153 negative (IHC staining×100). (G) ER negative (IHC staining ×100). (H) PR negative (IHC staining ×100). (I) c-erb-2 negative (IHC staining ×100).

**Figure 5:**
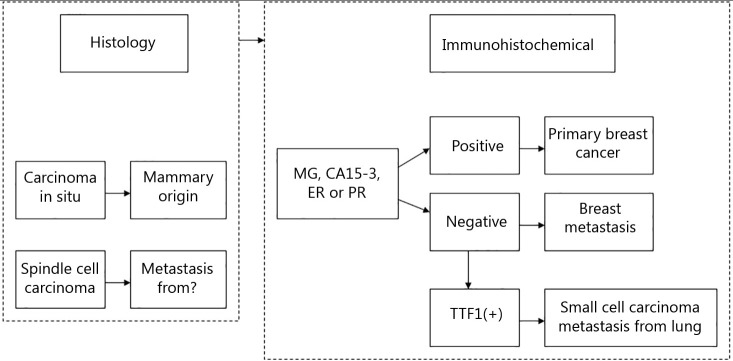
Differential diagnosis of case 2.

The H&E-stained paraffin sections of the breast specimen revealed carcinoma. The tumors were composed of densely packed hyperchromatic cells with scant cytoplasm and displayed an infiltrative growth pattern. Crush artifact and nuclear streaming occurred. Our differential diagnosis included primary breast carcinoma and metastatic carcinoma from the lungs. The tumor cells demonstrated immunoreactivity for TTF-1, NSE and CD56. The neoplastic cells lacked expression of MG, CA15-3, ER, PR, c-erb-2, EGFR, Syn, CgA and LCA (**Figure 4**). The expression of Ki67 and p53 showed strong nuclear staining in 60% and 80% of the tumor cells, respectively. No evidence of *in situ* carcinoma was observed. Small cell lung carcinoma metastasis was diagnosed based on histological and immunohistochemical staining patterns (**Figure 5**).

## Discussion

Patients with breast metastasis from pulmonary adenocarcinoma usually present a rapidly growing, painless, firm, well-circumscribed, and palpable mass with a predilection to the upper outer quadrant^1^^,^^6^^,^^7^^,^^12^. Unlike primary tumors, metastases do not generally demonstrate skin or nipple retraction despite their superficial location^4^^,^^12^. Distinguishing breast metastasis from primary mammary adenocarcinoma based on mammographic findings may be very difficult because of the wide range of imaging manifestations of metastatic lesions. Thus, metastasis can mimic a primary malignancy or even a benign breast tumor^3^^,^^4^^,^^8^. Mammography most commonly shows a well-defined rounded mass; however, multiple well-circumscribed lesions with smooth margins may also be presented^2^^,^^8^^,^^20^. Calcification is rare in breast metastases compared with metastases from serous papillary carcinoma of the ovary^8^^,^^20^^,^^23^.

We report two patients with breast metastases from pulmonary adenocarcinoma: one patient was characterized by neuroendocrine cells and diagnosed consistently with primary tumor; another patient exhibited small cell lung carcinoma. Patients with breast metastases from pulmonary adenocarcinoma usually present multiple, superficial, and well-circumscribed lesions. Pathologists should consider this diagnosis if morphological characteristics are unusual for a primary mammary tumor. About two-thirds of the cases possibly show histological characteristics indicative of possible metastasis. In one-third of such cases, medical history is vital for differential diagnosis. Small cell carcinoma suggests a pulmonary origin. Carcinoma in situ suggests a mammary origin^15^. The distinction between metastases from lung adenocarcinoma and primary mammary adenocarcinoma may cause a significant diagnostic dilemma. As such, the contribution of immunohistochemistry to a correct diagnosis is crucial. For example, TTF1 is a useful marker of pulmonary adenocarcinoma with a reported frequency of 68% to 80%; however, no stains are positively detected in breast carcinoma^25^^-^^27^ except a single case published by Klingen *et al*.^25^. The mammary origin is supported by the expression of ER, GCDFP-15 and MG. ER and GCDFP-15 are expressed in 80% and 45% to 53% of breast carcinoma, respectively^26^^,^^28^. Studies have revealed that ER expression in lung adenocarcinoma is low (7.6% to 14.1%)^26^^,^^29^. Furthermore, GCDFP-15 is expressed in 5.2% to 15% of lung adenocarcinoma^28^^,^^30^. MG is expressed in 48% to 72.1% of mammary adenocarcinoma but stained negatively in pulmonary adenocarcinoma^26^^,^^28^^,^^31^. Many cases of primary and metastatic breast adenocarcinoma (including ductal and lobular) are K7+/K20−/K5/6−, which exhibits an important diagnostic value in combination with ER, PR and GCDFP-15 immunohistochemistry in differentiating a primary breast adenocarcinoma from an adenocarcinoma at another primary site^32^. Lung adenocarcinomas are K7+ whereas lung squamous cell carcinomas are usually K7−^33^. Lung neuroendocrine carcinomas (large cell neuroendocrine carcinoma and small cell carcinoma) are usually negative for K7 and K20^34^. The expression of NSE, Syn or CgA is indicative of carcinomas with neuroendocrine differentiation^35^. Hepatocellular carcinomas generally express K8 and K18 but are negative for K7, K19 and K20^36^. Consequently, it is important to use a panel of antibodies as no single marker is completely sensitive or specific.

Scholars do not prefer mastectomy, although local excision and systemic treatment are recommended for breast metastasis. In general, metastasis to the breast has been associated with poor prognosis in patients who die within a year of diagnosis^6^. Our these two patients remained alive for 7 and 8 months after they were diagnosed with primary lung tumor and breast metastasis, respectively. Hence, patients should be followed up continually.

We presented two patients who developed metastasis to the breast from lung cancer. This metastasis is a very rare occurrence, and the prognosis of these patients is poor. A combination of clinical history, image data and pathology may provide effective and robust measures for disease diagnosis than any single modality. A detailed examination of the breast mass and specific immunohistochemical analysis are necessary to distinguish a primary breast cancer from metastasis to the breast to avoid unessential mastectomy and provide appropriate systemic treatment because the recommended therapy is possibly different with considerably varying outcomes.

## References

[1] Hajdu SI Urban JA Cancers metastatic to the breast. Cancer 1972;29:1691-1696 4337956 10.1002/1097-0142(197206)29:6<1691::aid-cncr2820290637>3.0.co;2-4

[2] Vizcaíno I Torregrosa A Higueras V Morote V Cremades A Torres V Metastasis to the breast from extramammary malignancies: a report of four cases and a review of literature. Eur Radiol 2001;11:1659-1665 11511887 10.1007/s003300000807

[3] Georgiannos SN Chin J Goode AW Sheaff M Secondary neoplasms of the breast: a survey of the 20th Century. Cancer 2001;92:2259-2266 11745279 10.1002/1097-0142(20011101)92:9<2259::aid-cncr1571>3.0.co;2-o

[4] Klingen TA Klaasen H Aas H Chen Y Akslen LA Secondary breast cancer: a 5-year population-based study with review of the literature. APMIS 2009;117:762-767 19775345 10.1111/j.1600-0463.2009.02529.x

[5] Sitzenfrey A. Mammakarzinom zwei jahre nach abdominaler radikal operation wegen doppelseitigen carcinoma ovarii. Prag Med Wochenschr 1907;32:221-235

[6] Williams SA Ehlers RA Hunt KK Yi M Kuerer HM Singletary SE Metastases to the breast from nonbreast solid neoplasms: presentation and determinants of survival. Cancer 2007;110:731-737 17582626 10.1002/cncr.22835

[7] Toombs BD Kalisher L Metastatic disease to the breast: clinical, pathologic, and radiographic features. AJR Am J Roentgenol 1977;129:673-676 409241 10.2214/ajr.129.4.673

[8] Noguera J Martínez-Miravete P Idoate F Díaz L Pina L Zornoza G Metastases to the breast: a review of 33 cases. Australas Radiol 2007;51:133-138 17419856 10.1111/j.1440-1673.2007.01681.x

[9] Bartella L Kaye J Perry NM Malhotra A Evans D Ryan D Metastases to the breast revisited: radiological-histopathological correlation. Clin Radiol 2003;58:524-531 12834635 10.1016/s0009-9260(03)00068-0

[10] Masmoudi A Mathieu MC Soria JC Breast metastasis from lung adenocarcinoma: a case report. Anticancer Res 2003;23:1825-1826 12820464

[11] Ramar K Pervez H Potti A Mehdi S. Breast metastasis from non-small-cell lung carcinoma. Med Oncol 2003;20:181-184 12835522 10.1385/MO:20:2:181

[12] Yeh CN Lin CH Chen MF Clinical and ultrasonographic characteristics of breast metastases from extramammary malignancies. Am Surg 2004;70:287-290 15098776

[13] Shukla R Pooja B Radhika S Nijhawan R Rajwanshi A. Fine-needle aspiration cytology of extramammary neoplasms metastatic to the breast. Diagn Cytopathol 2005;32:193-197 15754368 10.1002/dc.20198

[14] Gómez-Caro A Piñero A Roca MJ Torres J Ferri B Galindo PJ Surgical treatment of solitary metastasis in the male breast from non-small cell lung cancer. Breast J. 2006;12:366-367 16848849 10.1111/j.1075-122X.2006.00278.x

[15] Lee AH The histological diagnosis of metastases to the breast from extramammary malignancies. J Clin Pathol 2007;60:1333-1341 18042689 10.1136/jcp.2006.046078 PMC2095576

[16] Fulciniti F Losito S Botti G Di Mattia D La Mura A Pisano C Metastases to the breast: role of fine needle cytology samples. Our experience with nine cases in 2 years. Ann Oncol 2008;19:682-687 18048381 10.1093/annonc/mdm546

[17] Hsu W Sheen-Chen SM Wang JL Huang CC Ko SF Squamous cell lung carcinoma metastatic to the breast. Anticancer Res 2008;28:1299-1301 18505069

[18] Wood B Sterrett G Frost F Swarbrick N. Diagnosis of extramammary malignancy metastatic to the breast by fine needle biopsy. Pathology 2008;40:345-351 18446623 10.1080/00313020801911520

[19] Babu KS Roberts F Bryden F McCafferty A Downer P Hansell DT Metastases to breast from primary lung cancer. J Thorac Oncol 2009;4:540-542 19333072 10.1097/JTO.0b013e31819c8556

[20] Lee SK Kim WW Kim SH Hur SM Kim S Choi JH Characteristics of metastasis in the breast from extramammary malignancies. J Surg Oncol 2010;101:137-140 20082359 10.1002/jso.21453

[21] Maounis N Chorti M Legaki S Ellina E Emmanouilidou A Demonakou M Metastasis to the breast from an adenocarcinoma of the lung with extensive micropapillary component: a case report and review of the literature. Diagn Pathol 2010;5:82 21167048 10.1186/1746-1596-5-82 PMC3018363

[22] Yoon MY Song CS Seo MH Kim MJ Oh TY Jang UH A case of metachronous metastasis to the breast from non-small cell lung carcinoma. Cancer Res Treat 2010;42:172-175 20948923 10.4143/crt.2010.42.3.172 PMC2953781

[23] Ko K Ro JY Hong EK Lee S Micropapillary lung cancer with breast metastasis simulating primary breast cancer due to architectural distortion on images. Korean J Radiol 2012;13:249-253 22438695 10.3348/kjr.2012.13.2.249 PMC3303911

[24] Crona J Granberg D Norlén O Wärnberg F Stålberg P Hellman P Metastases from neuroendocrine tumors to the breast are more common than previously thought. A diagnostic pitfall? World J Surg 2013;37:1701-1706 23592057 10.1007/s00268-013-2037-2

[25] Klingen TA Chen Y Gundersen MD Aas H Westre B Sauer T Thyroid transcription factor-1 positive primary breast cancer: a case report with review of the literature. Diagn Pathol 2010;5:37 20565809 10.1186/1746-1596-5-37 PMC2896353

[26] Yang M Nonaka D. A study of immunohistochemical differential expression in pulmonary and mammary carcinomas. Mod Pathol 2010;23:654-661 20173733 10.1038/modpathol.2010.38

[27] Zamecnik J Kodet R. Value of thyroid transcription factor-1 and surfactant apoprotein A in the differential diagnosis of pulmonary carcinomas: a study of 109 cases. Virchows Arch 2002;440:353-361 11956814 10.1007/s00428-001-0552-2

[28] Takeda Y Tsuta K Shibuki Y Hoshino T Tochigi N Maeshima AM Analysis of expression patterns of breast cancer-specific markers (mammaglobin and gross cystic disease fluid protein 15) in lung and pleural tumors. Arch Pathol Lab Med 2008;132:239-243 18251583 10.5858/2008-132-239-AOEPOB

[29] Gomez-Fernandez C Mejias A Walker G Nadji M. Immunohistochemical expression of estrogen receptor in adenocarcinomas of the lung: the antibody factor. Appl Immunohistochem Mol Morphol 2010;18:137-141 19875957 10.1097/PAI.0b013e3181bec23b

[30] Striebel JM Dacic S Yousem SA Gross cystic disease fluid protein-(GCDFP-15): expression in primary lung adenocarcinoma. Am J Surg Pathol 2008;32:426-432 18300807 10.1097/PAS.0b013e318157a5a6

[31] Bhargava R Beriwal S Dabbs DJ Mammaglobin vs GCDFP-15: an immunohistologic validation survey for sensitivity and specificity. Am J Clin Pathol 2007;127:103-113 17145637 10.1309/TDP92PQLDE2HLEET

[32] Chu P Wu E Weiss LM Cytokeratin 7 and cytokeratin 20 expression in epithelial neoplasms: a survey of 435 cases. Mod Pathol 2000;13:962-972 11007036 10.1038/modpathol.3880175

[33] Nhung NV Mirejovský P Mirejovský T Melínová L Cytokeratins and lung carcinomas. Cesk Patol 1999;35:80-84 11038661

[34] Lyda MH Weiss LM Immunoreactivity for epithelial and neuroendocrine antibodies are useful in the differential diagnosis of lung carcinomas. Hum Pathol 2000;31:980-987 10987260 10.1053/hupa.2000.9076

[35] Segawa Y Takata S Fujii M Oze I Fujiwara Y Kato Y Immunohistochemical detection of neuroendocrine differentiation in non-small-cell lung cancer and its clinical implications. J Cancer Res Clin Oncol 2009;135:1055-1059 19152002 10.1007/s00432-009-0544-1 PMC12160139

[36] Hurlimann J Gardiol D. Immunohistochemistry in the differential diagnosis of liver carcinomas. Am J Surg Pathol 1991;15:280-288 1847609 10.1097/00000478-199103000-00008

